# Association of genetic polymorphisms CYP2A6*2 rs1801272 and CYP2A6*9 rs28399433 with tobacco-induced lung Cancer: case-control study in an Egyptian population

**DOI:** 10.1186/s12885-018-4342-5

**Published:** 2018-05-03

**Authors:** Nada Ezzeldin, Dalia El-Lebedy, Amira Darwish, Ahmed El Bastawisy, Shereen Hamdy Abd Elaziz, Mirhane Mohamed Hassan, Amal Saad-Hussein

**Affiliations:** 1 Chest Diseases, National Research Center, Cairo, Egypt; 2 Medical Oncology, National Cancer Institute, Cairo University, Cairo, Egypt; 3 Clinical Pathology, National Research Center, Cairo, Egypt; 4 Environmental Health & Preventive Medicine, National Research Center, Cairo, Egypt; 5 National Cancer Institute (NCI), Fom-Elkhalig Square, P.O.Box: 11796, Cairo, Egypt

**Keywords:** Lung cancer, CYP2A6, CYP2A6*2, CYP2A6*9, Polymorphism, Tobacco smoking, Nicotine metabolism, Egyptian

## Abstract

**Background:**

Several studies have reported the role of CYP2A6 genetic polymorphisms in smoking and lung cancer risk with some contradictory results in different populations. The purpose of the current study is to assess the contribution of the CYP2A6*2 rs1801272 and CYP2A6*9 rs28399433 gene polymorphisms and tobacco smoking in the risk of lung cancer in an Egyptian population.

**Methods:**

A case-control study was conducted on 150 lung cancer cases and 150 controls. All subjects were subjected to blood sampling for Extraction of genomic DNA and Genotyping of the *CYP2A6* gene SNPs (CYP2A6*2 (1799 T > A) rs1801272 and CYP2A6*9 (− 48 T > G) rs28399433 by Real time PCR.

**Results:**

AC and CC genotypes were detected in CYP2A6*9; and AT genotype in CYP2A6*2. The frequency of CYP2A6*2 and CYP2A6*9 were 0.7% and 3.7% respectively in the studied Egyptian population. All cancer cases with slow metabolizer variants were NSCLC. Non-smokers represented 71.4% of the *CYP2A6* variants. There was no statistical significant association between risk of lung cancer, smoking habits, heaviness of smoking and the different polymorphisms of *CYP2A6* genotypes.

**Conclusion:**

The frequency of slow metabolizers CYP2A6*2 and CYP2A6*9 are poor in the studied Egyptian population. Our findings did not suggest any association between *CYP2A6* genotypes and risk of lung cancer.

## Background

Lung Cancer is the most common cancer worldwide in term of new cases (1.8 million, 12.9%) & in term of death (1.6 million death, 19.4%) [1]. Cytochrome *P450 2A6 (CYP2A6)*, one of the forms of CYP expressed in the human respiratory tract, is the main enzyme involved in the metabolic activation of tobacco-specific nitrosamines to their carcinogenic forms [2]***.*** CYP2A6 inactivates nicotine, the principle component in cigarette smoke, to cotinine [3]. Thus it is reasonable to hypothesize that CYP2A6 activity may be related to the susceptibility of developing lung cancer among smokers.

The existence of a *CYP2A6* genetic polymorphism was suggested by evidence that there was extensive inter individual difference in the capacity of coumarin 7-hydroxylation [4, 5]***.*** Detecting the alleles of CYP2A6 can help us to describe different smoking behaviors and smoking-related diseases among individuals [6]. Numerous characterized alleles and some haplotypes that are uncharacterized have been identified & most of these are derived from single nucleotide polymorphisms (SNPS) in regulatory & coding regions [7]. The wild-type allele that is considered as a reference is CYP2A6*1A [8]. Currently CYP2A6*6, CYP2A6*7, CYP2A6*9, CYP2A6*10, CYP2A6*11, &, CYP2A6*13 are known to lead to reduced enzymatic activities while 5 variants (CYP2A6*2, CYP2A6*4, CYP2A6*5, CYP2A6*12, & CYP2A6*20) produce no functional enzyme [2, 9–12].

The CYP2A6*2 variant, appears to be one of the causal polymorphisms associated with decreased or virtually absent nicotine metabolism in Caucasians [13, 14]***.*** In East and Southeast Asian populations such as Chinese, Korean, Japanese, Malaysian and Thai, CYP2A6*2 is non-existent [15–19]. CYP2A6*4C is the most widely studied allele in all populations. East Asian populations such as Japanese, Chinese, Korean & malysian showed the highest frequency among populations (4.9–25.6%) [2, 12, 17, 20]. Caucasians had a frequency lower than 3% while Middle East populations such as Turkish and Iranian showed a lower frequency [21].CYP2A6*7 has a higher frequency in East and Southeast Asian populations [6, 17, 19, 22]. On the other hand, in Indian, African, Canadian native this allele isn’t found. It has also been reported in a lower frequency in Caucasian (≤0.3%) [21].

The CYP2A6*9 is present in all ethnic groups but its frequency varies from 6 to 8% in Europeans & Africans to 21% in Asian populations [23, 24]. Middle East population as Turkish [10] & Iranian [25] also showed a high frequency. CYP2A6*9 was reported as one of the most common variants of *CYP2A6* in Caucasians that modifies the levels of enzyme expression [10].

Considerable studies have reported the role of *CYP2A6* genetic polymorphisms in lung cancer risk with some contradictory results in different populations from ethnic variation [2, 26–30]. It seemed probable that genetic polymorphism in *CYP2A6* causing a lack of or reduced activity might result in lowering tobacco-induced lung cancer risk, by decreased smoking. This is due to decreased activation of carcinogens such as nitrosamines present in tobacco smoke, or by lower nicotine catabolism or by both [2, 6]*.*

Thus knowing that CYP2A6 activity affects genetic suscebility to tobacco- induced lung cancer, individuals possessing the CYP2A6*2 and/or CYP2A6*9, but not the CYP2A6*1A allele would be expected to be at lower risk for lung cancer.

We conducted a case-control study to assess the contribution of the CYP2A6*2 rs1801272 and CYP2A6*9 rs28399433 gene polymorphisms and tobacco smoking in the risk of lung cancer in an Egyptian population.

## Methods

### Subjects

This study was a collaboration between National Research Center and National Cancer Institute (NCI) Cairo University. A case-control study was conducted that included 150 unrelated adult patients with primary lung cancer and 150 unrelated controls. The case series consisted of patients presented to NCI details of which have been described elsewhere [31]. Almost half of the patients seen at NCI-Cairo came from the Cairo metropolitan area, whereas the rest of the patients were from other regions in South and North Egypt. Patients from the non-Cairo regions sought management at NCI-Cairo because it has the largest lung cancer services in Egypt for surgical and medical oncology & radiotherapy. All subjects included in the study were interviewed to fulfill questionnaire covering demographic information, data on smoking, occupational history and family history of malignancy. All subjects were aware of the study protocol and gave written informed consent. The study was approved by the ethical committee of the National Research Center.

#### DNA extraction

A peripheral blood sample is obtained from each subject through venipuncture. Blood was collected in tubes with EDETA. Extraction of genomic DNA was performed using a QIAamp DNA extraction kit according to the manufacturer’s protocol.

#### Selecting SNPs

We searched relevant publications from 1990 to 2013 in the National Center for Biotechnology Information (NCBI) data base using the following keywords: “CYP2A6”, “cigarette smoking”, “lung cancer” and “genetics”. Based on publication in Caucasian, African and Asian populations, we expect that: CYP2A6*2 and CYP2A6*9 would be interesting because the kinetics of nicotine metabolism are altered in individuals carrying any or both of these alleles.

#### Genotyping of *CYP2A6* gene

CYP2A6*2 and CYP2A6*9 polymorphisms were genotyped using TaqMan® SNP Genotyping Assays. Primers and probes were designed by Applied Biosystems (Foster City, CA, USA) and analyses were performed on ABI 7500 Real Time PCR system (Applied Biosystems) according to the manufacturer’s protocol.

For CYP2A6*2 (1799 T > A) [rs1801272; assay ID: C_27861808_60], the VIC/FAM sequence was as follows: CCCCTGCTCACCGCCAGTGCCCCGG[T/A]GGGCGTCGATGAGGAAGCCCGCCTC.

For CYP2A6*9 (− 48 T > G) [rs28399433; assay ID: C_30634332_10], the VIC/FAM sequence was as follows: GTGACGGCTGGGGTGGTTTGCCTTT[A/C]TACTGCCTGAAAAAGAGGGATGGAC.

For genotyping quality control, negative controls were included in all SNPs and 10% of samples were randomly selected and analyzed in duplicates and the concordance rate was 100%.

### Statistical analysis

Data were analyzed using SPSS version 18.0 (Chicago, IL, USA). Data were expressed as number and percentages of total for categorical variables. The Chi-square test (χ^2^) was used to compare the distribution of *CYP2A6* genotypes between the groups. Likelihood ratio was used when the expected count was less than 5 in more than 20% of the cells. The associations between genotype and risk of lung cancer were estimated by odds ratio (OR) and 95% confidence interval (95% CI) using logistic regression models. The ORs were adjusted for age, smoking status, and pack-years. *P*-value < 0.05 was considered significant.

## Results

Table 1 presents the baseline characteristics of 150 lung cancer cases and 150 controls. There were statistically significant differences between age & gender. The mean age of patients is (56.7 ± 9.79 years) and that of controls is (43.3 ± 11.1 years) (*p* <  0.001), denoting that older age is associated with higher risk of lung cancer. A statistically significant gender difference was found between the 2 groups; 48.7% males and 51.3% females in controls and 76% males and 24% females in cancer cases, (X^2^ = 25.82, *P* <  0.0001) with increased males risk to develop lung cancers than females.

**Table 1:** Characteristics of control and lung cancer cases and association between cigarette smoking and risk of lung cancer

	Control *n* = 150	Lung cancer *n* = 150	*P* value	
Age(years) Mean + SD	43.3 ± 11.1	56.7 ± 9.79	< 0.001	
Sex n(%)				
Male	73 (48.7%)	114 (76%)	< 0.0001	
Female	77 (51.3%)	36 (24%)		
Histological Types n (%)				
SCLC	20 (13.3%)			
NSCLC	130 (87.7%)			
Adenocarcinoma	73 (56.2%)			
Squamous cell carcinoma	18 (13.8%)			
Others^a^	39 (30%)			
	Control n (%)	Lung cancer n (%)	OR (95% C.I.)	*P* value
Smoking Status				
Smoker n (%)	30(20%)	93(62%)	2.78 (2.0–3.86)	0.0001
Non-smoker n (%)	120(80%)	57(38%)		
Pack-year				
< 20 n (%)	16(53.3%)	37(39.8%)	0.58 (0.25–1.32)	NS
> 20 n (%)	14(46.7%)	56(60.2%)		

*SCLC* small cell lung cancer, *NSCLC* non- small cell lung cancer

^a^Large cell carcinoma, undifferentiated, spindle, mucinous, Broncho alveolar carcinoma, carcinoid, sarcomatoid carcinoma, anaplastic

Smoking was significantly higher among cancer cases (62% smokers) compared to control (20% smokers), *P* <  0.0001, and (OR = 2.78, CI: 2.0–3.86) denoting a higher risk to develop lung cancer in smokers by 2.78 times the non-smokers, independent of the heaviness of smoking.

**Fig. 1:**
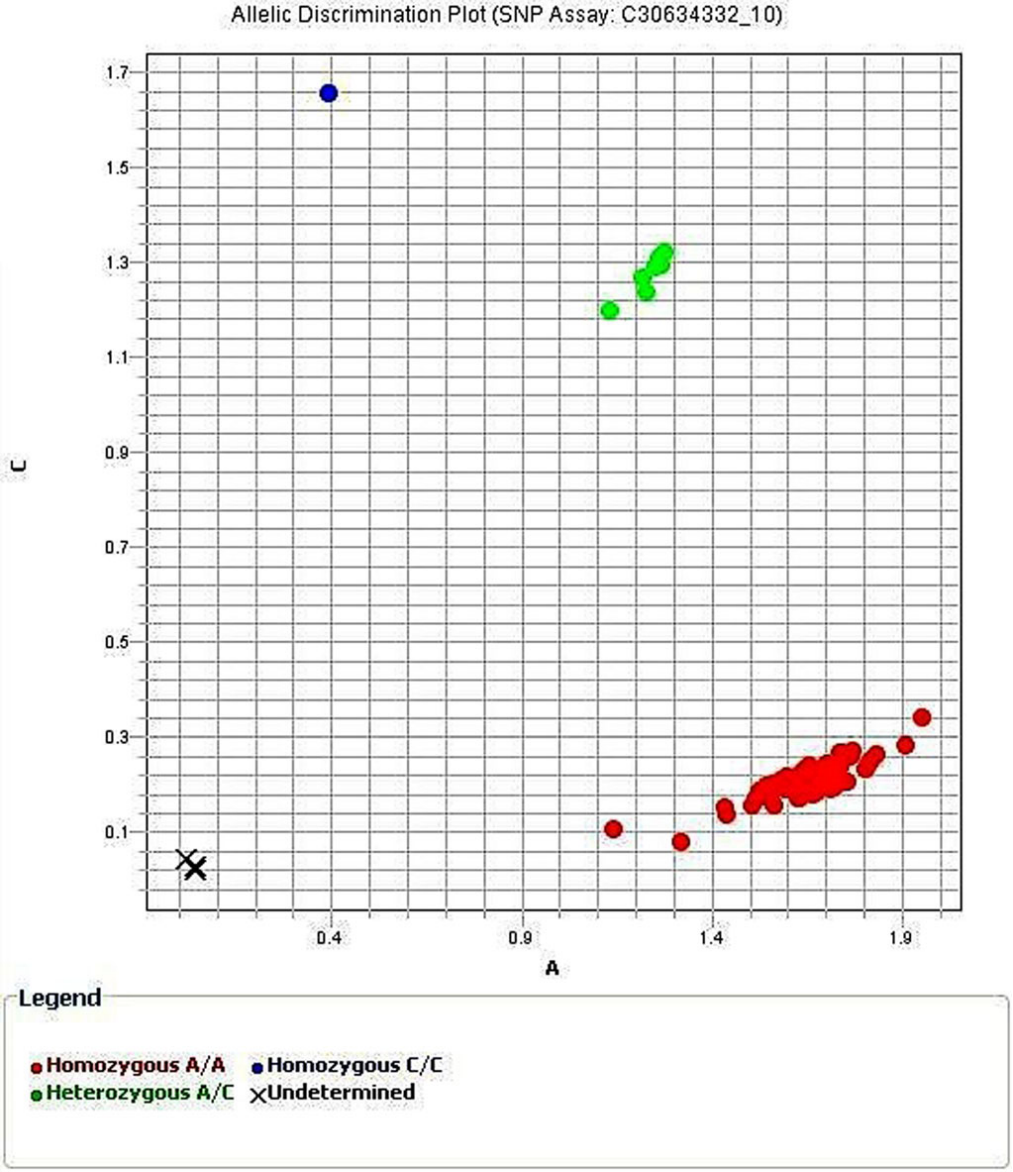
Allelic discrimination plot of CYP2A6*9 rs28399433 genotypes

**Fig. 2:**
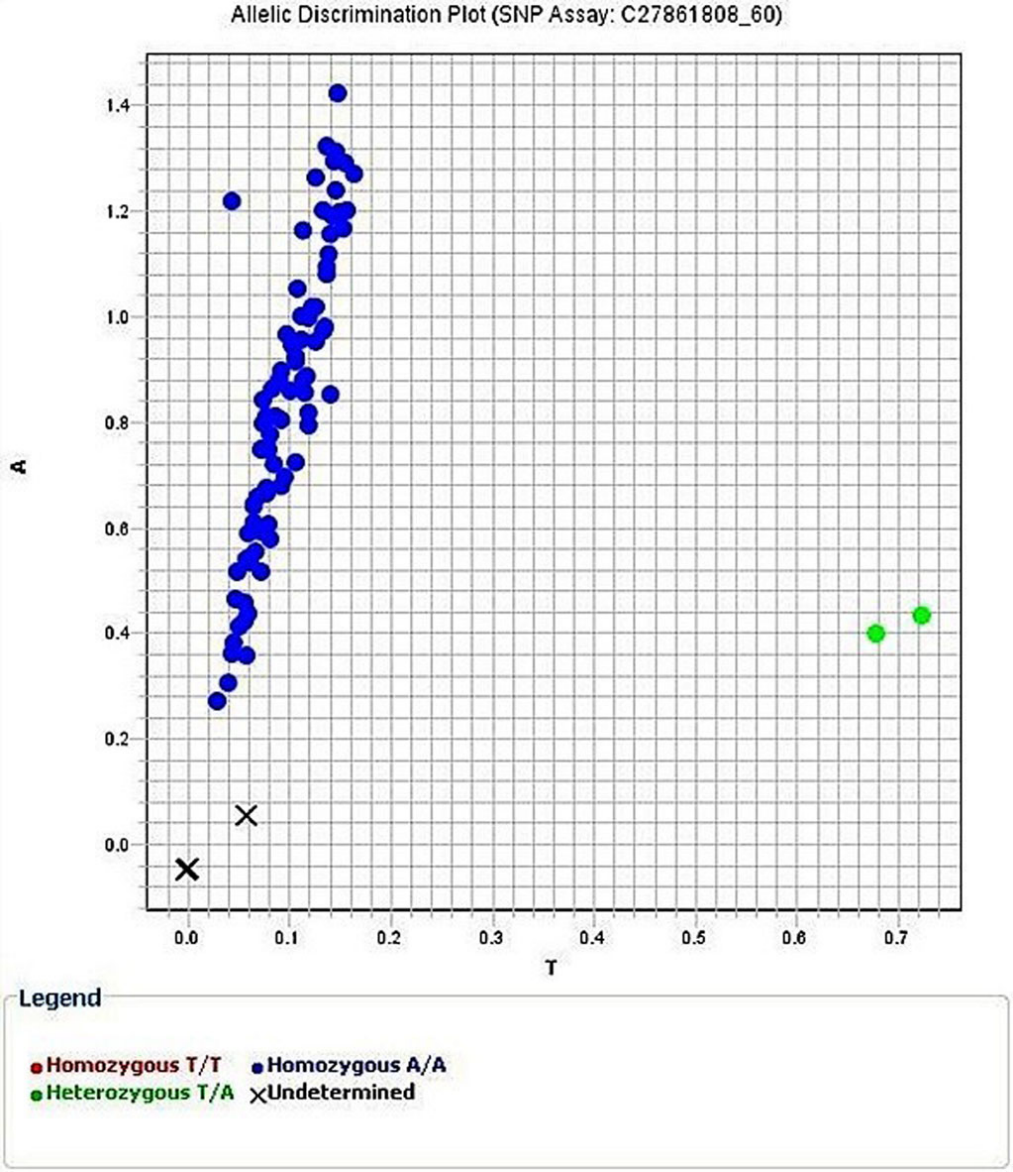
Allelic discrimination plot of CYP2A6*2 rs1801272 genotypes

Two genotypes were detected in CYP2A6*9; heterozygote *AC* and homozygote *CC.* ***AC*** genotype was found in 4 cases in control group; 3 males and 1 female, all cases were non-smokers and 6 cases in lung cancer group; 4 males and 2 females from which 2 males were smokers > 20 pack-year. All cases were diagnosed as NSCLC (2 adenocarcinoma, 1 squamous and 3 others). *CC* genotype was found in 1 control case, a male, smoking > 20 pack-year (Fig. 1). One heterozygous genotype was detected in CYP2A6*2; AT. It was found in one case of lung cancer; a non-smoker male diagnosed as adenocarcinoma (Fig. 2). The 2 polymorphisms CYP2A6*9 AC and CYP2A6*2 AT were detected in a non-smoker female control case.

There was no statistical difference between the Wild type CYP2A6*1AA and CYP2A6*9, CYP2A6*2 polymorphisms regarding age, sex, smoking status, pack-year (Table 2). There was no statistical significant association between risk of lung cancer, smoking habits, heaviness of smoking and the different polymorphisms of *CYP2A6* genotypes (Table 3).

**Table 2:** Frequency of *CYP2A6* genotypes in the studied Egyptian population

Total *n* = 300	CYP2A6*1 **AA** ***n*** **=** **287** ^**a**^	CYP2A6*9 *n*=12^a^	*P*-value	CYP2A6*2 *n*=2^a^	*P*-value
Age(years)					
Mean + SD	**49.8 ± 12.3**	51.6 ± 13.3	0.34	45.5 ± 6.4	0.62
Sex					
Male n (%)	**178 (62%)**	7 (63.6%)	0.74	1 (50%)	0.72
Female n (%)	**109 (38%)**	4 (36.4%)		1 (50%)	
Smoking Status					
Smoker n (%)	**120 (41.8%)**	3 (18.2%)	0.15	0 (0%)	0.15
Non-smoker n (%)	**167 (58.2%)**	9 (81.8%)		2 (100%)	
Pack-year					
< 20 n (%)	**53 (44.2%)**	0 (0%)	0.17	0 (0%)	
≥ 20 n (%)	**67 (55.8%)**	3 (100%)			

^a^One case had the 2 polymorphisms was added to both number of cases of CYP2A6*9 and CYP2A6*2

**Table 3:** *CYP2A6* genotypes, cigarette smoking and risk of lung cancer in the studied Egyptian population

	CYP2A6		
	CYP2A6*1 *(AA)*	CYP2A6*9 *(AC)*	CYP2A6*2 *(AT)*
Control/ cancer n	144/143	5/6	1/1
OR (95% C.I.)	1.2 (0.36–4.02)	0.8 (0.2–2.8)	0.99 (0.1–16)
*P* value	*P* = 0.77	*P* = 0.77	*P* = 0.99
Non-Smoker			
Control/ cancer n	114/52	5/4	1/1
OR (95% C.I.)	1.7 (0.4–6.7)	0.6 (0.1–2.2)	2.1 (0.1–34.6)
* P* value	*P* = 0.42	P = 0.42	*P* = 0.60
Smoker			
Control/ cancer n	30/91	0/2	(a)
OR (95% C.I.)	1.0 (0.99–1.1)	1.0 (1.0–1.1)	
*P* value	*P* = 0.30	P = 0.30	
Pack-year < 20			
Control/ cancer n	16/37		
OR (95% C.I.)	(a)	(a)	(a)
* P* value			
Pack-year ≥20			
Control/ cancer n	14/54	0/2	(a)
OR (95% C.I.)	1.0 (0.99–1.09)	1.0 (0.99–1.1)	
P value	*P* = 0.36	P = 0.36	

OR of the CYP2A6*9 (CC) cannot be calculated as it is one case only. (a): cannot be calculated there is no cases in one of the two groups

There was no statistical significant difference in the *CYP2A6* genotypes in different stages of lung cancer (Table 4).

**Table 4:** Effects of CYP2A6 genotypes on Stages of lung cancer in 150 lung cancer cases

Genotypes of *CYP2A6* *n* = 150		Stage			Likelihood	*P*-value
		II *n* = 25	III *n* = 44	IV *n* = 81		
CYP2A6*1 AA	143 (95.3%)	23 (92%)	43 (97.7%)	77 (95.1%)	2.25	0.69
CYP2A6*9 AC	6 (4.0%)	2 (8%)	1 (2.3%)	3 (3.7%)		
CYP2A6*2 AT	1 (0.7%)	0 (0.0%)	0 (0.0%)	1 (1.2%)		

## Discussion

*CYP2A6* has been proposed as a novel target for smoking cessation because of its major contribution to nicotine metabolism***.*** To the best of our knowledge this is the first study to address the relationship between CYP2A6 status, smoking & lung cancer in an Egyptian population. Our data are valuable because cases that presented to NCI were from different governorates of Egypt. The CYP2A6*1A allele is considered the wild type, corresponding to normal enzymatic activity [16]***.*** Based on reports in the literature, we expect that CYP2A6*2 & CYP2A6*9 would be intresting because they code for reduced activity. Many human nicotine pharmacokinetic & CYP2A6 studies have demonstrated the contribution of multiple decrease-, increase-, and loss-of-function polymorphisms in CYP2A6 to the variability in nicotine metabolism [23]. In our study the control group was sampled at random thus their age distribution was much younger than that of lung cancer cases. This explains the statistical significant difference in age between cases and control. Age isn’t a confounder in our study and isn’t intended to be examined in the analysis of CYP2A6 polymorphism. The tested polymorphisms are not affected by age and our aim was to to assess the contribution of the CYP2A6*2 and CYP2A6*9 gene polymorphisms and tobacco smoking in the risk of lung cancer.

### Frequency and ethnic variation

Our results showed that the frequency of CYP2A6*2 and CYP2A6*9 were 0.7% and 3.7% respectively. There was no statististical significant difference in the frequency of the tested variants between control and lung cancer goups. Further statistical studies were difficult due to low frequency of variants. Neverthelss, these poor frequencies were reported in other studies. In African populations (Afro American & Ethiopian), the CYP2A6*2 frequency is less than 1% [16, 17, 19] while it was found to be one of the major variant alleles in Caucasians; with frequency 1–3% [17, 19]. The frequency of CYP2A6*2, and *9 detected in a Brazilian population was 1.7, and 5.7% respectively [32] while in Turkish population the CYP2A6* 2 is not existing [33]. Recent researches that had analysed different frequencies of CYP2A6 genetic variants in different ethnic groups; found that the frequency of CYP2A6*2, and *9 in Caucasian almost double our findings in the Egyptian population [23]. The frequencies of CYP2A6 *2 allele in different ethenic population were 1.1%, 0.0%, 2.2%, 0.0%, 0.0% and those of CYP2A6*9 were 7.1%, 15.5%, 7.1%, 15.6%, 20.3% in African American, Canadian, Caucasian, Chinese and Japanese respectively [9, 10, 12, 18, 19, 34–36].

### Smoking

Many studies have concluded that *CYP2A6* slow inactivator, or absent enzyme activity genotypes are associated with lower risk for smoking, altered smoking intensity, fewer cigarettes smoked, and increased quitting [2, 13, 19, 20, 37, 38]. Nevertheless, other studies are in disagreement with these conclusions [26, 39, 40]***.***

Little is known about the genetic factors contributing to smoking in Egyptians. In the present study, although no statistical difference were found between *CYP2A6* variants and smoking status, most cases with *CYP2A6* polymorphisms were non-smoker, 10 cases from 13; 100% and 81.8% non-smokers in CYP2A6*2 and CYP2A6*9 respectively compared to 58% in CYP2A6*1 indicating a role of CYP2A6 gene variants in affecting smoking status, with slow metabolizers smoking less than fast metabolizers. Verde & colleagues [41]***,*** found that CYP2A6*2 polymorphism was strongly linked to smoking status. Moreover, it was found that the amounts of cigarette consumed daily by individuals, who harbored CYP2A6*9, were significantly less than that consumed by those who carried CYP2A6*1 (*P* = 0.01) [6]***.*** Slow metabolizers (as determined by CYP2A6*2, *4, *9 and *12 alleles), are very unlikely to be smokers, take smaller puff volumes and smoke fewer cigarettes per day. They also have lower levels of dependence, benefit more from nicotine patch replacement therapy and are more capable to quit compared to normal metabolizers [2, 16, 19, 38, 42–48]***.***

Contrastingly two different meta-analyses failed to confirm any evidence of the association between the *CYP2A6* diminished-activity polymorphisms and cigarette consumption. The investigators of both concluded that there is no association between the reduced-activity *CYP2A6* alleles and the number of cigarettes smoked [49, 50]*.*

Malaiyandi & colleagues [16] have postulated that the continued effect of slow metabolism on reducing the number of cigarette smoked, allthrough the smoking history of slow inactivators, may influence withdrawal mechanisms and consequently aids quitting among these individuals. The noticeable temporal effect of *CYP2A6* on smoking behavior and nicotine dependence necessitates further research. The poor frequencies of the variant alleles of slow inactivators extend our understanding of the impact of *CYP2A6* genotype on smoking risk and behavior in Egyptian, and have important implications for smoking aetiology. The number of smokers in Egypt is 13 million out of a population of 90 million according to statistics by the state-run Central Agency for Public Mobilization and Statistics. There is minimal change in the number of smokers inspite of 40% tax increments since 2010 or photos of damaged lungs placed on every pack of cigarettes as a warning from the hazardous health consequences of smoking [51] (Mansour, 2013). According to the study by Loffredo & colleagues [52], the national prevalence of former cigarette smoking among males was 18.1%, and 27.5% reported current smoking***.***

### Lung cancer

Sqamous cell carcinoma and Small cell lung cancer (SCLC) are major types of lung cancer caused by smoking, while adenocarcinoma isn’t regarded as a common histological type in smokers [53, 54]*.* In the present study**,** all lung cancer cases (6)**,** with CYP2A6*2 and *9 polymorphisms were diagnosed as NSCLC, 3 of them were adenocarcinoma. Statistical interpretations could not be done due to the low frequency of variants in the study. A former study [29]*,* did not find a significant association between this *CYP2A6* polymorphism and risk of adenocarcinoma or other histological types of lung cancer.

Studies of *CYP2A6* inhibition demonstrated a role for the enzyme in the occurrence of lung cancer and hence have directed the interest in *CYP2A6* as a target for cancer prevention [28, 55]*.* Investigating the role of *CYP2A6* genotype in effective programs implemented for lung cancer screening may improve cancer risk prediction and assist selecting individuals who could benefit from preventative measures whether behavioural or pharmacological [56]*.* Ariyoshi & colleagues [2]***,*** demonstrated that individuals homozygous for the CYP2A6*1A allele (*1A/*1A) have the highest risk for tobacco-related lung cancer in Japanese male smokers. They also concluded that due to higher prevelence of the functionally active *CYP2A6* gene in Caucasians compared to Asians, the genetic polymorphism of *CYP2A6* may be a factor justifying the interindividual difference in predisposition to lung cancer among smokers. Consistent to the previous concept Benowitz et al. [57] concluded that the slower nicotine metabolism and therefore lower nicotine uptake per cigarette may explain lower incidence of lung cancer in Asians compared to Caucasians***.*** In the present study we failed to find a significant association between *CYP2A6* genotypes and risk of lung cancer. Our data is similar to the study by London & colleagues [39] who reported no significant relationship between the *CYP2A6* inactive allele, mainly concentrating on the CYP2A6*2, and lung cancer risk.

Discordant results have been demonstrated on the association of *CYP2A6* genetic polymorphisms and lung cancer risk [2, 26, 27, 29, 39, 58]. These contradictory results seem to be caused by the too small frequencies of the inactive alleles such as CYP2A6*2 and CYP2A6*4 in some studies to find a possible relationship with adequate statistical power [26, 29]. The protecting effect of *CYP2A6* slow metabolism on lung cancer risk is more obvious in populations including subjects common to harbour *CYP2A6* non-functional alleles as the Japanese compared to Caucasian & other populations who include individuals harbouring low frequency of *CYP2A6* slow metabolizers. It is unclear whether the risk for lung cancer is mediated through the effect on smoking behavior or whether it also involves increased sensitivity to carcinogens present in cigarette smoke [59]. Liu & colleagues [60] suggested that decreasing or abolishing the activity of the *CYP2A6* by means of specific inhibitors might prevent the frequency of occurrence of tobacco-induced lung cancer in smokers only***.*** In this study, 5 out of 7 lung cancer cases (71.4%) with *CYP2A6* slow metabolizer were non- smokers.

It is worth noting that our study has some limitations. First because the number of subjects is low, significant differences in smoking behaviors were not observed between those with and without CYP2A6*2 & CYP2A6*9. Further studies with larger population samples may present more detailed results. Second, we concentrated on only CYP2A6*2 & CYP2A6*9 genetic polymorphisms. The effects of other polymorphisms of *CYP2A6* on smoking habits should be examined; CYP2A6*4 and *7 are also major functional polymorphisms.

## Conclusions

Our study, in the setting of patients with lung cancer demonstrates that the frequency of slow metabolizer CYP2A6*2 and CYP2A6*9 are poor in the studied Egyptian population; who are non-smokers. Our findings did not suggest any association between *CYP2A6* genotypes and risk of lung cancer.

## Abbreviations

CI: Confidence interval
*CYP2A6*: Cytochrome *P450 2A6*
NCBI: National Center for Biotechnology Information
NCI: National Cancer Institute
NSCLC: Non small cell lung cancer
OR: Odds ratio
SCLC: Small cell lung cancer
SD: Standard deviation
SNPs: Single nucleotide polymorphisms
